# High-Reliability Signal Quality Validation for Biosignals Using Sensor Fusion and Software Indices

**DOI:** 10.3390/s26113478

**Published:** 2026-06-01

**Authors:** Basel Adams

**Affiliations:** Fraunhofer Institute for Reliability and Microintegration, Technische Universität Berlin, 13355 Berlin, Germany; basel.adams@tu-berlin.de

**Keywords:** signal quality assessment, biosignals, electrocardiogram, motion artifact detection, lead-off detection, hybrid validation framework, dynamic time warping, signal-to-noise ratio, baseline wander, sensor integrity screening, signal quality index evaluation, accelerometer-based artifact rejection

## Abstract

This paper proposes a two-stage hybrid framework for biosignal quality validation that produces beat-level or segment-level labels for real-time filtering and offline dataset curation. The framework is quantitatively validated exclusively on ECG data. Its modular architecture is designed to extend to further non-stationary periodic biomedical time-series signals including photoplethysmography (PPG), impedance cardiography (ICG), phonocardiography (PCG), electromyography (EMG), and electroencephalography (EEG) through modality-specific parameter adaptation; however, this broader applicability currently reflects architectural extensibility rather than experimentally validated performance. A prerequisite is synchronized acquisition of the primary biosignal together with inertial motion sensing (IMU/accelerometer) and electrode impedance or lead-off status, with the IMU positioned near the sensing electrodes. The first stage performs sensor-integrity gating to reject intervals corrupted by motion or poor electrode contact. The second stage applies software signal quality indices to the remaining beats, including physiological plausibility constraints (R to R peaks analysis), DTW-based morphological consistency against adaptive templates, frequency domain SNR estimation, and baseline wander quantification. This study systematically evaluates and compares the classification performance of six complementary sensor-level and software-based signal quality assessment methods. When integrated within the proposed hybrid framework, validation against expert-annotated ECG quality labels from 20 healthy participants demonstrates high methodological classification accuracy (98.1%), achieving approximately a 98% F1-score, 99% sensitivity, and 97% specificity. Prospective validation on patient populations with cardiovascular pathology is identified as a necessary step toward clinical deployment. This modular approach improves the reliability of downstream analysis by preventing corrupted data from entering feature extraction and model training pipelines, enabling more stable physiological monitoring in free-living conditions, reducing false alarms in continuous monitoring applications, and generating higher-quality datasets for AI-based diagnostic systems.

## 1. Introduction

Wearable biosignal monitoring enables continuous physiological assessment outside clinical settings and supports applications such as rhythm screening, longitudinal monitoring, and remote patient management. However, real-world recordings are frequently degraded by motion artifacts, baseline drift, intermittent electrode contact, and environmental interference, which can compromise diagnostic reliability, increase false alarms, and contaminate datasets used for machine learning model development.

Most existing signal quality approaches operate purely in the software domain and evaluate the waveform after acquisition, which can waste computation on irrecoverably corrupted intervals and often yields binary accept/reject outputs rather than granular labels suitable for dataset curation. Conversely, hardware-level indicators such as accelerometer-derived motion measures and electrode impedance/lead-off monitoring enable early rejection, but alone cannot capture all corruption sources (e.g., EMG interference or residual waveform distortion). This motivates a unified framework that combines hardware-informed screening with robust software indices to provide beat-level, interpretable quality outcomes suitable for real-time and offline use.

This work presents a two-stage signal quality validation framework integrating (i) sensor integrity gating via synchronized IMU and impedance/lead-off monitoring, and (ii) complementary software-based SQI evaluation. Outputs are fused into a configurable global quality score (1 = unusable to 6 = excellent) supporting artifact rejection, feature extraction, and AI dataset curation. While the architecture is designed to generalize across beat-structured modalities (ECG, PPG, ICG) and segment-based signals (EMG, EEG, EOG), experimental validation in this work is conducted on ECG data, which provides expert-annotated ground truth and well-established quality benchmarks.

This work constitutes a methodological validation study, establishing the framework’s signal quality discrimination performance under controlled wearable conditions with diverse motion artifact and electrode contact scenarios. Prospective clinical validation on patient populations is identified as a subsequent step. This work makes four key contributions to biosignal quality assessment:

- Weighted fusion of hardware and software indices delivering interpretable ordinal quality labels (1–6) rather than binary accept/reject outputs.
- Configurable weight coefficients enable systematic adaptation for different applications. For example, arrhythmia-aware monitoring can disable RR-interval checks while prioritizing morphological consistency, or resource-constrained devices can reduce computational load by downweighting expensive DTW analysis without requiring architectural changes.
- The framework achieves 98.13% accuracy, 98.81% sensitivity, and 96.70% specificity on 8644 expert-annotated ECG beats—a 7.89 percentage-point gain over the strongest individual metric and superior to six established benchmarks—while maintaining critically low false positive rates essential for high-confidence AI dataset curation

The framework targets diverse populations, ranging from healthy individuals using fitness trackers to patients requiring continuous cardiac monitoring, remote heart failure assessment, or post-operative care. It addresses both real-time monitoring enabling automated artifact rejection and adaptive alarms and offline dataset curation for retrospective analysis and machine learning model development. Critically, the granular quality scores enable systematic verification and filtering of data used for training AI diagnostic models, ensuring that only high-quality segments are included in training, validation datasets and increase clinician confidence in wearable monitoring technology for patient care.

## 2. Related Work

Biosignal quality assessment in wearable monitoring has emerged as a critical challenge, requiring approaches that balance computational efficiency with robustness across diverse real-world conditions. This work extends the signal quality validation methodology from the AI-enhanced smart textile system for cardiovascular monitoring [1], which demonstrated the necessity of robust quality assessment for reliable multimodal biosignal analysis. Prior work in this domain can be organized into three complementary categories: hardware-centric approaches leveraging auxiliary sensors, software-based signal quality indices, and recently developed hybrid frameworks that integrate both modalities.

Hardware-level quality assessment employs auxiliary sensors to identify corrupted signal segments before computationally intensive algorithmic processing occurs. Accelerometer-based motion artifact detection has become widely adopted in wearable ECG systems, with An et al. demonstrating that adaptive filtering using synchronized impedance pneumography signals as motion reference can effectively reduce motion-induced artifacts in ambulatory ECG recordings [2]. Electrode–skin impedance monitoring has emerged as a complementary approach, where continuous or periodic impedance measurements identify poor contact quality [3]. Such hardware-based methods offer significant computational advantages through early rejection of irrecoverably corrupted beats, reducing downstream processing burden. However, hardware indicators alone cannot capture signal corruption arising from electromagnetic interference, muscle artifacts, or physiological variations, necessitating integration with software-based validation techniques.

Software quality assessment methods analyze the acquired biosignal waveform itself to quantify its integrity across multiple dimensions. Classical approaches employ R-peak detection reliability as a quality indicator, with the Pan-Tompkins algorithm serving as both a feature extractor and implicit quality assessor [4]. Physiological plausibility metrics, particularly RR interval analysis, have been extensively employed to identify arrhythmias and artifact-corrupted segments, spanning from simple range checking to comprehensive signal quality index frameworks incorporating multi-source data fusion [5]. Morphological quality assessment using template matching has been investigated through various approaches. Odinaka et al. proposed template matching combined with physiological feasibility constraints for ECG quality assessment in textile-based wearable systems [6]; Locatelli et al. validated a straightforward algorithm for wearable ECG quality evaluation in clinical settings [7]; and Dynamic Time Warping (DTW), which accommodates temporal variations in beat morphology, has been applied to pulsatile signal quality assessment by Li and Clifford [8]. Additionally, Krasteva et al. demonstrated real-time arrhythmia detection combined with ECG quality monitoring in intensive care settings [9]. Frequency-domain approaches computing signal-to-noise ratio or power spectral density have proven effective for identifying high-frequency noise and baseline wander [10,11]. Orphanidou et al. proposed a comprehensive signal quality index framework deriving and validating morphological and statistical indicators for ambulatory ECG and PPG monitoring in wireless wearable systems [11]. Despite their interpretability advantages, purely software-based methods incur computational costs on fundamentally unusable data and lack the early rejection capability that hardware information provides.

Recent work has begun integrating hardware and software quality indicators to leverage complementary strengths. Neri et al. provided a comprehensive review of wearable ECG monitoring devices and AI-enabled diagnostic capabilities, demonstrating the growing integration of acquisition and processing pipelines in ambulatory settings [12]. Li and Boulanger proposed an automatic method combining empirical mode decomposition with adaptive filtering to suppress baseline wander and motion artifacts in ambulatory ECG signals, providing an efficient preprocessing step before software quality indices [13]. Machine learning-based quality classifiers have emerged, trained on expert-annotated datasets to learn quality patterns from combined features [14,15]; Smital et al. specifically demonstrated real-time quality assessment for long-term wearable ECG in free-living conditions [14], achieving practical deployment on resource-constrained platforms. However, such approaches often require substantial labeled data and frequently function as black boxes, limiting interpretability for clinical deployment and regulatory approval.

Quality assessment methods have been developed for biosignal types beyond ECG. Satija et al. provide a comprehensive review of signal processing techniques for ECG quality assessment surveying a broad range of existing approaches [15]. For PPG signals, Elgendi proposed an optimal signal quality index specifically tailored to photoplethysmogram waveform characteristics [16]. Impedance cardiography quality assessment has focused on signal enhancement and noise rejection; Escalona et al. proposed recursive signal averaging combined with multi-stage wavelet denoising to improve arm–ICG signal quality for long-term wearable cardiac contractility monitoring [17]. Phonocardiography quality evaluation uses spectral features and heart sound segmentation with probabilistic state-based models [18]. EMG quality assessment emphasizes frequency domain analysis and amplitude distribution [19], while EEG quality assessment relies on automatic artifact detection methods; Arpaia et al. provided a systematic review of techniques for artifact detection in wearable EEG, covering both classical and deep learning approaches [20]. These modality-specific approaches, while effective, lack a unified framework that could be efficiently adapted across different biosignals.

Despite this substantial body of work, critical gaps remain. Most existing methods provide binary classifications rather than granular quality scores suitable for dataset curation and weighted analysis. Existing approaches lack modularity across biosignal types, requiring complete independent redesign instead of modality-specific parameter adaptation. Validation against expert-labeled ground truth across diverse real-world conditions remains limited, and synchronized IMU and electrode impedance data have not been systematically formulated as explicit design prerequisites for comprehensive quality validation. This paper addresses these gaps through a modular two-stage architecture with configurable fusion weighting, granular ordinal quality scoring (1–6 scale), and explicit incorporation of synchronized auxiliary sensor data as foundational design elements.

## 3. Methodology

**Figure 1:**
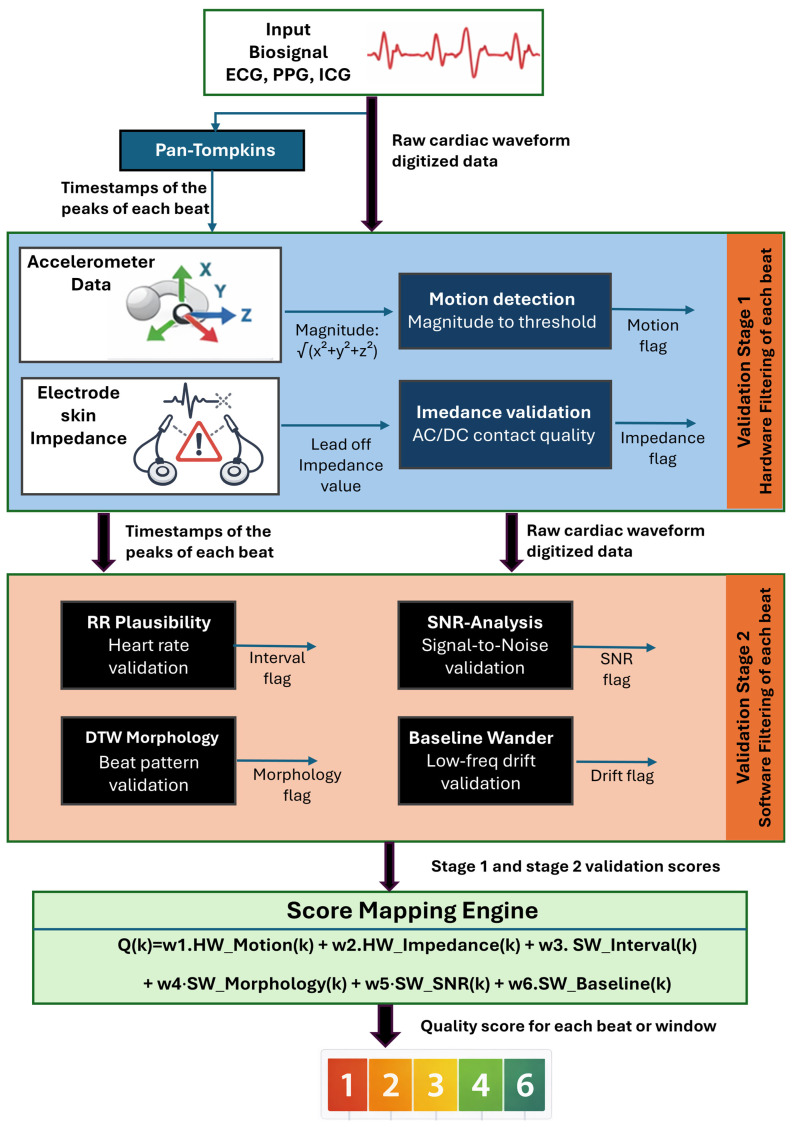
Two-stage biosignal quality assessment framework showing the sequential gating of beats through Stage I hardware-based filtering (motion and impedance detection) and Stage II software quality indices.

The proposed method is a two-stage signal quality validation framework for the real-time and offline assessment of biosignals, validated in this work on ECG as the reference modality, as illustrated in Figure 1. All quantitative results reported in this paper are based exclusively on ECG data. Descriptions of PPG, ICG, EMG, and EEG throughout this section reflect the intended design scope and theoretical applicability of each module; modality-specific quantitative validation is deferred to future work.

This method generates a beat-level for cardiac based signals (or segment-level in case of EEG or EMG signals) quality labels ranging from 1 (unusable) to 6 (excellent) by combining hardware-informed gating with software-based signal quality indices. This hybrid architecture enables early rejection of irrecoverably corrupted data while maintaining computational efficiency.

The framework assumes synchronized acquisition of the primary biosignal (e.g., ECG, PPG, or ICG), a three-axis accelerometer (IMU) positioned near the electrodes for motion supervision, and electrode impedance or lead-off status for contact integrity monitoring. All channels are time-aligned at acquisition as described in [1]. Figure 1 summarizes the sequential validation stages applied to each detected beat.

### 3.1. Initial Preprocessing

As an initial preprocessing step, cardiac beats are segmented using robust fiducial point detection. For ECG signals, R-peaks are identified with the classical Pan–Tompkins QRS detection algorithm [4], which applies bandpass filtering and nonlinear transformations to enhance QRS complexes while suppressing baseline drift and high-frequency noise. The detected R-peak timestamps define consecutive cardiac cycle boundaries, enabling beat-synchronous application of all subsequent hardware- and software-based assessment indices. For applications requiring full waveform delineation including P- and T-wave boundaries, multiscale parameter estimation methods provide robust fiducial point detection across diverse noise conditions [21].

For PPG and ICG recordings, systolic peaks serve as the primary segmentation landmarks. This approach is motivated by the higher detection reliability of R-peaks and systolic peaks compared with other fiducial features (e.g., P- or T-waves in the case of ECG), which often exhibit lower signal-to-noise ratios and greater inter-subject variability. Anchoring quality assessment to these stable landmarks minimizes segmentation error propagation and ensures consistent beat-level evaluation across diverse waveform morphologies and recording conditions.

### 3.2. Stage I: Hardware Based Sensor Integrity Filtering

#### 3.2.1. Motion Artifact Detection

Motion artifacts are among the most significant sources of signal degradation in wearable biosignal monitoring, particularly for ECG, EEG, ICG and PPG recordings. To enable the early rejection of motion-corrupted cardiac cycles, the proposed framework incorporates synchronized tri-axial inertial measurement unit (IMU) data as a hardware-level quality indicator.

Let $a_{x}[n]$, $a_{y}[n]$, and $a_{z}[n]$ denote the raw accelerometer channels acquired concurrently with the primary biosignal. As a first preprocessing step, an orientation-independent acceleration magnitude is computed over the entire raw IMU recording:

$$m[n]=\sqrt{a_{x}[n{]}^{2}+a_{y}[n{]}^{2}+a_{z}[n{]}^{2}}$$

**Figure 2:**
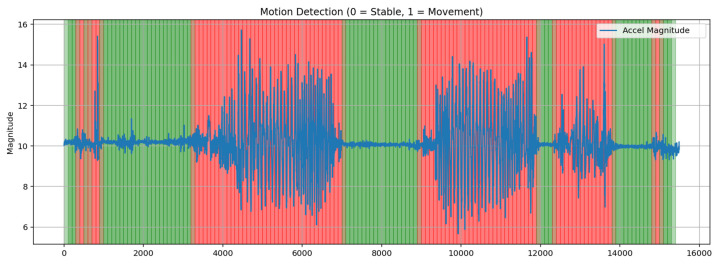
IMU-based motion detection module.

This transformation provides a scalar motion representation that is robust to sensor rotation and enables consistent motion quantification independent of device orientation. Figure 2 illustrates the IMU-based motion detection module, where green regions indicate stable intervals with minimal motion corresponding to beats suitable for further processing, and red regions indicate movement-contaminated intervals where motion artifacts are expected to degrade biosignal waveform quality.

Following beat segmentation (e.g., using R-peak detection for ECG or the systolic peak detection for PPG and ICG), the acceleration magnitude signal m[n] is evaluated within each individual cardiac beat interval.

For the k-beat window $B_{k}=[n_{k},n_{k+1}]$, defined by two consecutive detected peaks, a beat-specific motion index is computed as the variance in the acceleration magnitude during that beat:

$$\sigma_{k}^{2}=Var\left(m[n_{k}:n_{k+1}]\right)$$

This measure reflects the degree of movement or sensor displacement occurring specifically during the acquisition of the corresponding biosignal beat.

Each beat is assigned to a binary hardware motion flag based on a predefined motion threshold $\tau_{\mathrm{motion}}$ which varies depending on the sensor used and its placement:

$$HW_{\mathrm{Motion}}(k)=\left\{\begin{array}{cc} 0, & \sigma_{k}^{2}>\tau_{\mathrm{motion}}\;(\mathrm{motion}\text{-}\mathrm{corrupted}\mathrm{beat})\\ 1, & \sigma_{k}^{2}\leq \tau_{\mathrm{motion}\;}(\mathrm{acceptable}\mathrm{beat}) \end{array}\right.$$

Beats located in the time range of movement exceeding the threshold are marked as unreliable due to the high likelihood of motion-induced artifacts contaminating the waveform morphology.

#### 3.2.2. Electrode Lead-Off Impedance Verification

Intermittent electrode detachment and unstable skin–electrode coupling represent major sources of signal corruption in wearable electrophysiological monitoring. Poor contact conditions introduce abrupt baseline shifts, high impedance noise, and complete signal dropout, which can severely compromise the reliability of downstream feature extraction and diagnostic interpretation. Therefore, the proposed framework incorporates electrode lead-off and impedance monitoring as a second hardware-level validation mechanism.

For surface biopotential recordings such as ECG, EMG, and EEG, electrode contact quality can be assessed through lead-off detection, which determines whether an electrode is properly connected to the skin. Most modern analog front-end (AFE) integrated circuits for electrophysiological sensing (e.g., ECG/EEG/EMG chips) provide built-in lead-off detection functionality, enabling continuous or periodic monitoring of electrode integrity without additional external circuitry.

Lead-off detection is commonly implemented using either DC-based or AC-based impedance measurement techniques. In DC lead-off detection, a small bias current or voltage is applied to the electrode, and the resulting electrode potential is monitored. When the electrode becomes disconnected, the input node saturates toward supply rails, producing a detectable voltage shift. DC methods are computationally simple and are widely used in low-power wearable ECG systems. In contrast, AC lead-off detection injects a small high-frequency test signal (outside the physiological band, e.g., several kHz) and estimates the electrode–skin impedance from the measured response. Because the excitation frequency is separated from the biosignal spectrum, AC methods provide a more robust and physiologically independent estimate of electrode contact quality.

While lead-off detection is naturally available for electrode-based biopotential measurements, modalities such as photoplethysmography (PPG) require alternative strategies.

PPG sensors rely on optical coupling and not electrode contact; therefore, classical impedance lead-off detection is not applicable. Instead, contact quality can be inferred through surrogate indicators such as photodiode DC level monitoring (optical saturation or poor coupling) or perfusion index (pulse amplitude reduction).

In the proposed two-stage validation framework, impedance or lead-off status is evaluated synchronously with each detected beat interval. Beats exhibiting impedance values **Z** outside an acceptable operating range, or showing abrupt impedance transitions indicative of transient contact loss, are assigned a rejected hardware contact flag:

$$HW_{\mathrm{Impedance}}(k)=\left\{\begin{array}{cc} 0, & Z_{k}>[Z_{min}]\;(\mathrm{poor}\mathrm{contact})\\ 1, & Z_{k}<[Z_{min}]\;(\mathrm{acceptable}\mathrm{contact}) \end{array}\right.$$

In the case of ECG, for instance, the beat is labeled as “Pass” when the measured electrode–skin impedance remains below the upper bound **Z-min** = 10 kΩ, consistent with standard electrode contact guidelines per Webster [3] and the AFE hardware specification of the acquisition platform used in this study [1].

By combining motion-derived IMU gating with impedance-based electrode contact validation, the proposed Stage I hardware filtering ensures that only beats acquired under stable mechanical and electrical conditions are subjected to computationally heavier software-based quality indices. This hybrid strategy improves robustness in free-living wearable environments, reduces false acceptance of corrupted beats, and enhances the trustworthiness of biosignal datasets used for AI-driven diagnostics.

### 3.3. Stage II: Software-Based Signal Quality Index Evaluation

Beyond temporal plausibility constraints, high-quality cardiac biosignals are expected to exhibit consistent beat morphology across successive cardiac cycles. Even when motion artifacts and electrode contact failures are excluded in Stage I, residual waveform corruption may still arise from electromagnetic interference, EMG muscle noise added to ECG, or partial sensor displacement.

Stage II applies a set of complementary software-based signal quality indices (SQIs) directly to the biosignal waveform to assess its physiological consistency, morphological integrity, and noise characteristics.

Depending on the intended application, the input to Stage II may consist either of (i) cardiac beats that have successfully passed the Stage I hardware gating in real-time operation, or (ii) the full raw biosignal recording in offline post-processing scenarios where all segments are retained for retrospective inspection. This flexibility enables the framework to support both computationally efficient real-time filtering and comprehensive dataset curation.

#### 3.3.1. Physiological Plausibility (RR Interval Analysis)

A widely used software-based signal quality indicator for cardiac biosignals is the analysis of beat-to-beat interval consistency. Physiological plausibility constraints on peak-to-peak intervals and R-peak amplitude stability have been employed in multiple prior works to detect spurious peak detections and artifact-contaminated segments, particularly R to R times and amplitudes in ECG monitoring and wearable quality assessment pipelines [13]. Such approaches rely on the observation that under stable physiological conditions, successive RR intervals evolve smoothly, whereas abrupt deviations are often caused by motion artifacts, noise-triggered false peaks, or missed detections.

**Figure 3:**
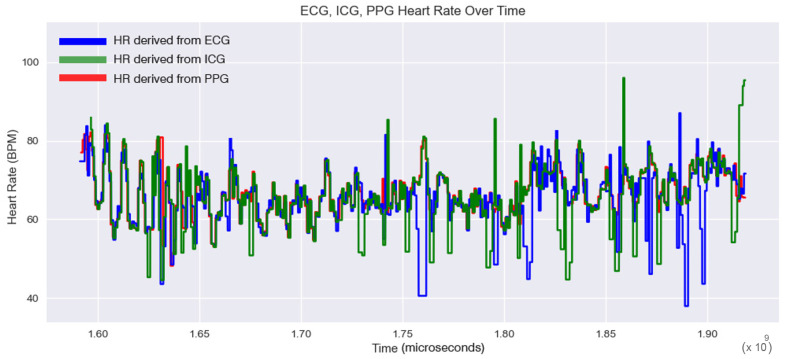
Heart rate estimated simultaneously from ECG, ICG, and PPG recordings using the multimodal acquisition platform described in [1], shown here for contextual illustration of the synchronized acquisition consistency. Quantitative validation in this study is based exclusively on ECG data. Red ranges indicate intervals of high beat-to-beat variability associated with poor signal quality.

Figure 3 illustrates the heart rate trajectories derived simultaneously from the recorded ECG, ICG, and PPG signals. All three modalities exhibit comparable heart rate trends over time, confirming the consistency of the synchronized multimodal acquisition. Minor deviations between the sensor-derived heart rate estimates are primarily attributable to modality-specific noise and transient recording artifacts, which affect peak detection accuracy in individual channels during wearable measurements. This figure is illustrative and demonstrates the conceptual applicability of the motion detection module across modalities; quantitative validation in this study was performed exclusively on ECG data.

Following peak detection (e.g., Pan–Tompkins for ECG or systolic peak detection for PPG/ICG), the RR interval of the k-th beat is computed as

$$RR_{k}=t_{k}-t_{k-1}$$

where $t_{k}$ denotes the timestamp of the current peak detected. To assess plausibility, the current interval is compared against a short-term reference distribution derived from a moving window of recent valid RR intervals. Let $RR_{\mathrm{median}}$ denote the median value within this window. A beat is considered physiologically implausible if

1. The RR interval falls outside predefined physiological bounds $RR_{min}\;and\;RR_{max}$ (e.g., 50 bpm to 250 bpm), or;
2. The deviation from the recent **R-R median** exceeds a relative tolerance threshold.

The resulting physiological quality flag is defined as

$$SW_{\mathrm{Phys}}(k)=\left\{\begin{array}{cc} 0, & RR_{k}\notin [RR_{min},RR_{max}]\;\mathrm{or}\;|RR_{k}-RR_{\mathrm{median}}|\;>0.5\;RR_{\mathrm{median}}\\ 1, & \mathrm{otherwise} \end{array}\right.$$

The RR interval–based plausibility metric is specific to beat-structured cardiac signals and is not applicable to non-cardiac biosignals such as EEG, EMG, or EOG, where no meaningful beat-to-beat timing exists. In arrhythmia-oriented applications including atrial fibrillation, premature ventricular contractions, ventricular tachycardia, and other conduction abnormalities, RR intervals inherently exhibit pathological irregularity that does not reflect poor signal quality. Applying the **SW_Phys** interval criterion in such contexts would incorrectly reject clinically meaningful beats and introduce systematic bias into downstream rhythm analysis. Therefore, the RR interval plausibility flag is conditionally disabled when the monitoring application is arrhythmia-aware, and physiological plausibility validation is in this case restricted exclusively to R-peak amplitude stability assessment (**F_amp**), which reflects electrode contact and signal integrity independently of rhythm regularity.

Let $A_{i}$ denote the amplitude of the detected R-peak corresponding to beat i. R-peak amplitude variations beyond expected physiological limits may indicate transient electrode detachment or motion-induced contact instability.

For each beat, the detected R-peak amplitude is compared against a short-term reference distribution derived from a sliding window of previously accepted beats. Let $\tilde{A}$ denote the median R-peak amplitude within this reference window. A beat is considered amplitude-plausible if $A_{i}$ lies within predefined physiological amplitude bounds, and the deviation from the reference median does not exceed a predefined relative tolerance threshold.

Formally, the amplitude-based physiological plausibility flag is defined as

$$F_{\mathrm{amp}}(i)=\left\{\begin{array}{cc} 1, & \mathrm{if}\;|A_{i}-\tilde{A}|\;\leq \delta_{A}\\ 0, & \mathrm{otherwise} \end{array}\right.$$

where $\delta_{A}$ represents an empirically calibrated tolerance parameter reflecting acceptable amplitude variability.

#### 3.3.2. Morphological Consistency Using Multi Directional DTW Pattern Matching

High-quality cardiac biosignals are expected to exhibit consistent waveform morphology across successive cardiac cycles. This validation step is particularly meaningful for beat-structured periodic biosignals, where each cardiac cycle contains characteristic waveform components that can be compared against an expected physiological template.

Morphological similarity is quantified using Dynamic Time Warping (DTW), a well-established alignment technique that measures the distance between two time-series sequences while allowing non-linear temporal stretching and compression. Unlike pointwise Euclidean distance or correlation-based measures, DTW is robust to small variations in beat duration and local temporal shifts, which commonly occur in wearable physiological recordings due to heart rate variability or sensor sampling jitter.

Moreover, Dynamic Time Warping (DTW) was selected for morphological validation instead of cross-correlation, since cardiac beat timing naturally varies due to local stretching and compression, and DTW provides robust alignment under such temporal variability.

**Figure 4:**
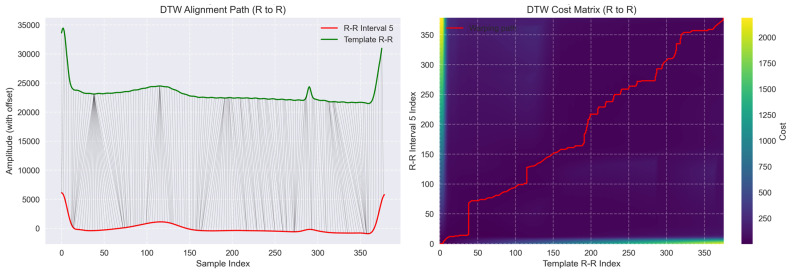
DTW-based morphological ECG beat-to-template comparison, showing strong alignment with the reference waveform.

**Figure 5:**
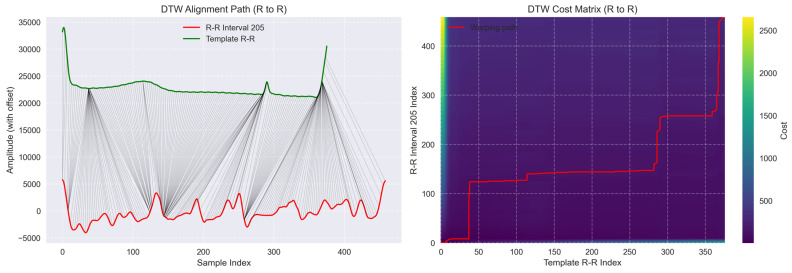
DTW-based morphological ECG beat-to-template comparison, showing no alignment with the reference waveform.

Figure 4 illustrates a “pass” example of DTW-based beat-to-template validation. The candidate beat waveform is aligned to a reference template through an optimal warping path, and the resulting DTW cost matrix provides an interpretable representation of morphological similarity. A near-linear warping trajectory within the matrix indicates strong waveform consistency, whereas irregular deviations from the diagonal as in Figure 5 suggest distortion or artifact contamination.

Given a candidate beat waveform $x_{i}$ and a reference template T, **DTW** computes an optimal alignment path P that minimizes the accumulated cost:

$$DTW(x_{i},T)=\underset{P}{min}\sum_{(m,n)\in P}d(x_{i}(m),T(n))$$

where P denotes the warping path between both sequences.

In the proposed framework, reference templates are constructed adaptively using a running average of previously accepted high-quality beats. For ECG signals, rhythm-specific templates (e.g., sinus rhythm, premature ventricular contractions, ventricular tachycardia, ventricular fibrillation) may also be incorporated to avoid penalizing clinically meaningful morphological variations.

To further enhance robustness, the statistical distribution of DTW distances is analyzed across consecutive beats. Specifically, the mean μ and standard deviation σ of the accumulated DTW cost values are computed to assess the stability and linearity of the warping path within the cost matrix. Under normal physiological conditions, morphologically consistent beats produce DTW distances with low variance, yielding a compact distribution around the mean as in Figure 4. In contrast, artifact-corrupted beats introduce irregular alignment paths and nonlinear deviations, resulting in increased standard deviation, as shown in Figure 5.

Accordingly, a beat is classified as morphologically inconsistent if its **DTW** distance deviates significantly from the expected distribution:

$$DTW(x_{i},T)>\mu +k\sigma$$

where k is an empirically calibrated tolerance parameter.

Finally, a beat is accepted as morphologically consistent if its **DTW** similarity score satisfies the predefined morphology threshold:

$$Q_{morph}(i)=\left\{\begin{array}{cc} 1, & DTW(x_{i},T)\leq \theta_{DTW}\\ 0, & \mathrm{otherwise} \end{array}\right.$$

where $\theta_{DTW}$ represents the morphology acceptance threshold.

This beat-level morphology flag provides an interpretable indicator of waveform integrity and supports rejection of subtle distortions that may not be captured through hardware gating alone.

DTW-based morphological validation is primarily suited for cardiac pulse-like biosignals (ECG, PPG, ICG), where individual beats form meaningful repeatable units. For non-beat-centric biosignals such as EMG or EEG, waveform morphology is not strictly repetitive and therefore template-based DTW validation is less appropriate.

#### 3.3.3. Signal-to-Noise Ratio (SNR)

While heart rate and morphological plausibility metrics assess the structural consistency of individual cardiac cycles, signal quality is also strongly influenced by broadband noise contamination. The signal-to-noise ratio (SNR)-based validation in frequency-domain is an excellent quality assessment that has been widely adopted in biosignal validation literature, particularly for ambulatory ECG and PPG recordings, where spectral power characteristics provide robust discrimination between clean physiological activity and artifact-dominated segments.

In the proposed framework, frequency-domain SNR and baseline wander metrics were selected instead of wavelet-based methods due to their substantially lower computational complexity, enabling efficient real-time implementation on resource-constrained wearable platforms. Furthermore, SNR-based evaluation was preferred over zero-crossing analysis because beat-structured biosignals such as ECG, PPG, and ICG exhibit well-defined spectral characteristics, allowing physiologically meaningful band separation. In contrast, zero-crossing methods ignore signal magnitude information and are highly sensitive to baseline wander, which can significantly distort crossing counts and reduce reliability in ambulatory recordings. Rahman et al. evaluated the robustness of ECG signal quality indices across diverse noise conditions, demonstrating that SNR-based metrics provide stable discrimination performance under realistic wearable operating conditions [10].

For each detected beat window $x_{k}[n]$, the power spectral density (**PSD**) is estimated using standard Fourier-based approaches, such as Welch’s method with a Hann window. The spectral energy is then separated into a physiological signal band and a noise-dominated band. Specifically, the signal power is computed by integrating the **PSD** over the dominant cardiac frequency range:

$$P_{\mathrm{signal}}=\int_{f_{1}}^{f_{2}}PSD\left(f\right)\;df\;\mathrm{e.g.},\;f_{1}=1\;\mathrm{Hz},\;f_{2}=39\;\mathrm{Hz}$$

In contrast, the noise power is estimated by integrating over an out-of-band region dominated by high-frequency artifacts:

$$P_{\mathrm{noise}}=\int_{f_{3}}^{f_{4}}PSD\left(f\right)\;df\;\mathrm{e.g.},\;f_{3}=40\;\mathrm{Hz},\;f_{4}=1\;\mathrm{kHz},$$

The beat-level **SNR** is then defined as

$$SNR_{k}\;(dB)=10{log}_{10}\left(\frac{P_{\mathrm{signal}}}{P_{\mathrm{noise}}}\right)$$

Higher **SNR** values correspond to cleaner beats with well-preserved morphology, whereas low **SNR** values indicate significant noise contamination.

To support interpretable quality scoring, the **SNR** value is converted into a binary software quality flag:

$$SW_{\mathrm{SNR}}(k)=\left\{\begin{array}{cc} 1, & SNR_{k}\geq \theta_{\mathrm{SNR}}\\ 0, & SNR_{k}<\theta_{\mathrm{SNR}} \end{array}\right.$$

where $\theta_{\mathrm{SNR}}\;$ is a calibrated threshold (e.g., 10 dB), depending on modality and application requirements.

Typical interpretations are

- 20–50 dB: clinical-grade, high-quality signal;
- 10–20 dB: acceptable quality for monitoring;
- 0–10 dB: noisy waveform with reduced diagnostic reliability.

#### 3.3.4. Baseline Wander Assessment

In addition to high-frequency noise contamination, wearable cardiac biosignals are frequently affected by low-frequency baseline distortions. Baseline wander is primarily caused by respiration, slow body movements, electrode polarization effects, and gradual sensor displacement. Such drift can significantly distort waveform morphology, reduce the reliability of peak detection, and bias downstream feature extraction.

Following the baseline wander quantification approach proposed in [13], baseline wander is quantified in the frequency domain by measuring the relative contribution of very low-frequency, e.g., 0 to 0.5 Hz, spectral power. For each detected beat window $x_{k}[n]$, the power spectral density PSD(f) is estimated using Welch’s method. The baseline power is then computed by integrating the **PSD** over the baseline drift band:

$$P_{\mathrm{baseline}}=\int_{0}^{1}PSD(f)\;df$$

This frequency range captures slow oscillations and drift components that are not part of the physiological cardiac waveform. In parallel, the total physiological signal power is computed over the main ECG bandwidth:

$$P_{\mathrm{total}}=\int_{0}^{40}PSD(f)\;df$$

The baseline signal quality index is then defined as the normalized baseline-relative power:

$$basSQI_{k}=1-\frac{P_{\mathrm{baseline}}}{P_{\mathrm{total}}}$$

This formulation yields values close to 1 for signals with minimal baseline drift, while lower values indicate increasing dominance of baseline wander.

To support interpretable beat-level quality scoring, the baseline SQI is converted into a binary validation flag:

$$SW_{\mathrm{Baseline}}(k)=\left\{\begin{array}{cc} 1, & basSQI_{k}\geq \theta_{\mathrm{baseline}}\\ 0, & basSQI_{k}<\theta_{\mathrm{baseline}} \end{array}\right.$$

where **θ_baseline_** = 0.75 in this study, empirically calibrated on the independent calibration dataset as detailed in Section 3.5, and corresponds to a maximum tolerated baseline power contribution of 25% of total signal power.

**Figure 6:**
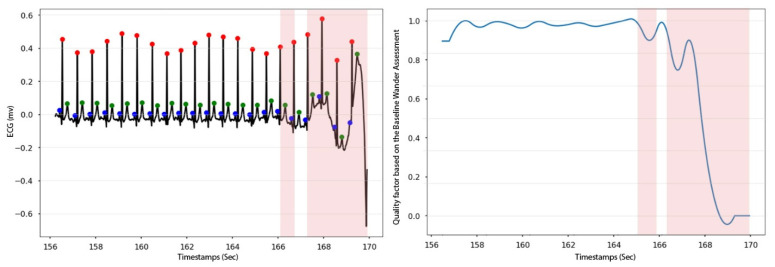
Baseline wander quality assessment results. Red areas indicate intervals where the baseline quality factor falls below the acceptance threshold and the beat is classified as rejected.

Figure 6 shows the results of the baseline wander quality assessment. The left panel shows an ECG segment with detected beat landmarks and baseline drift. The right panel presents the corresponding baseline quality factor, which remains high during stable periods and drops sharply as low-frequency baseline wander dominates the signal, indicating degraded beat quality.

### 3.4. Global Quality Factor Computation

To obtain a unified measure of beat-level signal integrity, the binary validation flags produced by Stage I and Stage II are fused into a single global quality score using a weighted linear model.

For each detected beat k, the framework produces the following six binary indicators:

**Stage I (Hardware): HW_Motion(k), HW_Impedance(k);**

**Stage II (Software): SW_Interval(k), SW_Morphology(k), SW_SNR(k), SW_Baseline(k).**

Each indicator takes a value of 1 if the corresponding criterion is passed and 0 if it is failed. These indicators are combined as follows:

$$\begin{array}{l} Q(k)=w_{1}\cdot HW_{\mathrm{Motion}}(k)+w_{2}\cdot HW_{\mathrm{Impedance}}(k)+w_{3}\cdot SW_{\mathrm{Interval}}(k)+w_{4}\cdot SW_{\mathrm{Morphology}}(k)+w_{5}\\ \;\;\;\;\;\;\cdot SW_{\mathrm{SNR}}(k)+w_{6}\cdot SW_{\mathrm{Baseline}}(k) \end{array}$$

The weighting coefficients wi are constrained to sum to exactly 5:

Σ **wi** = 5, i = 1,…,6

This constraint ensures that **Q(k)** ∈ [0, 5], where **Q(k)** = 0 indicates that all six validation criteria failed and **Q(k)** = 5 indicates that all criteria passed. The final beat-level quality score is then obtained directly as

**Score(k) = 1 + Q(k)**

yielding a scale from 1 (unusable) to 6 (excellent) with no additional mapping step required.

The weights applied in this study for ECG validation are given in Table 1. They were determined on the independent calibration dataset described in Section 3.5 (900 beats from three participants not included in the evaluation cohort), with each weight assigned proportionally to the relative discriminative contribution of the corresponding individual flag as reflected in its individual accuracy on the calibration dataset. Higher weights are assigned to indices with stronger individual discrimination, and lower weights to indices with weaker individual discrimination or conditional applicability.

For the binary classification performance evaluation reported in Section 5—accuracy, sensitivity, specificity and F1-score—beats with **Score(k)** ≥ 4 (**Q(k)** ≥ 3) are classified as Pass, meaning the majority of the weighted criteria are classified as Pass, while beats with **Score(k)** < 4 are classified as Fail. This threshold was selected on the independent calibration dataset (Section 3.5) as the operating point that maximized the F1-score against the expert-annotated ground truth labels, and it was fixed prior to evaluation on the 20-participant evaluation set.

The weighting coefficients and the Pass/Fail threshold are application-configurable parameters. For arrhythmia-aware applications, w3 is set to 0 and the remaining weights are rescaled proportionally to restore the sum-to-5 constraint, ensuring that the quality score range and mapping remain consistent. Weights may similarly be adapted for different signal modalities, electrode configurations, or computational constraints without requiring structural modification of the pipeline.

### 3.5. Threshold Specification and Parameter Selection

All threshold parameters used in the framework were determined prior to performance evaluation using a combination of established physiological reference ranges from the literature and empirical calibration on an independent held-out dataset. The calibration dataset comprised 900 expert-annotated ECG beats recorded from three participants who were not included among the 20 participants used for performance evaluation. These calibration recordings were acquired using the same hardware platform and experimental protocol described in Section 4, ensuring hardware-consistent but participant-independent parameter selection. Empirically calibrated parameters—specifically **τ_motion**, k (DTW sigma multiplier), and **θ_baseline**—were optimized on this calibration subset by maximizing the F1-score against expert-annotated ground truth labels. The fusion weights (Table 1) were assigned proportionally to the relative discriminative contribution of each individual flag as reflected in its standalone accuracy on the calibration dataset. The Pass/Fail threshold (Score ≥ 4) was likewise selected on the calibration subset as the operating point that maximized F1-score. All parameters were fixed after calibration and were not adjusted on the 20-participant evaluation dataset. No participant contributed data to both the calibration and evaluation datasets.

Table 2 summarizes all threshold parameters, their values as applied in this study, and the basis for their selection.

All thresholds were fixed before evaluation and were not adjusted post hoc. The framework’s modular architecture allows these values to be recalibrated for different sensor hardware, electrode configurations, or target populations without modifying the pipeline structure.

It should be noted that all reported parameter values are specific to the hardware and experimental setup used in this study [1], and may require recalibration depending on factors such as IMU sensor type and placement, electrode material and geometry, lead-off detection method (DC- vs. AC-based), skin–electrode interface characteristics, and target application or patient population.

## 4. Data Acquisition and Experimental Protocol

All biosignal recordings analyzed in this study were acquired using the smart textile wearable monitoring system described in [1], which enables synchronous high-resolution multimodal cardiovascular sensing under realistic wearable conditions. The platform was configured to simultaneously record 12-lead ECG with integrated AC-based electrode lead-off detection, impedance cardiography (ICG), photoplethysmography (PPG), and inertial measurement unit (IMU) signals for motion supervision and many other cardiac diagnostic related signals. All modalities were sampled synchronously at 1 kHz, ensuring precise beat-level alignment across channels.

A dedicated multimodal dataset was collected from 20 different participants (13 males and 7 females) without cardiac disorders to capture inter-subject variability in waveform morphology and artifact conditions. All data were securely stored via the Fraunhofer IZM OwnCloud infrastructure for offline analysis.

All procedures were approved by the Ethics Committee of the Department of Psychology and Ergonomics at Technical University of Berlin, and the study was classified as ethically unobjectionable under tracking number 2865545.

Cardiac cycles were segmented using detected peak timestamps (Lead II R-peaks for ECG). Each extracted ECG beat was manually annotated as Pass (physiologically interpretable waveform with minimal artifacts) or Fail (corrupted by motion, electrode detachment, noise, or baseline drift). These annotations served as the ground truth for evaluating the proposed two-stage quality validation framework.

Across all datasets, a total of 8644 beats were analyzed, comprising 6153 Pass beats [71%] and 2491 Fail beats [29%], reflecting the natural class imbalance typical in wearable monitoring environments. All 8644 beats from the 20 evaluation participants were used exclusively for performance evaluation. Framework thresholds and fusion weights were calibrated on an independent dataset of 900 beats from three separate participants not included in the evaluation cohort (Section 3.5) and were fixed prior to evaluation. No threshold was tuned on evaluation outcomes, and no iterative optimization procedure was applied.

## 5. Results

**Figure 7:**
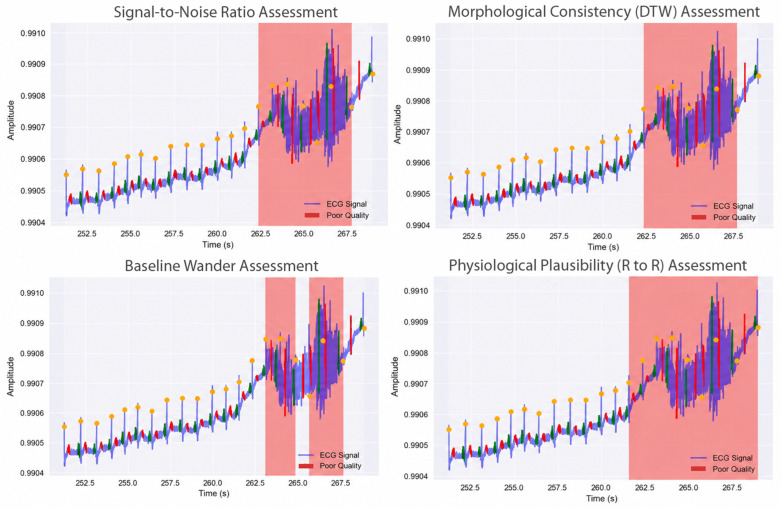
Stage II signal quality assessment results. Stage II signal quality assessment results. Red areas indicate intervals of poor signal quality where one or more validation criteria failed.

The behavior of the four software-based SQI components during a representative ECG segment containing clean and motion-corrupted intervals (red shaded regions) is illustrated in Figure 7. The ECG waveform remains stable during the clean period, while significant distortion and variability appear in the poor-quality segment. The SNR metric captures increased high-frequency noise, DTW reflects reduced morphological consistency, baseline assessment identifies low-frequency drift, and the R–R plausibility check detects irregular beat intervals caused by artifact-related misdetections.

Table 3 presents the statistical summary of accuracy of all stages across the 20 ECG datasets and implicitly constitutes a–n ablation analysis of the framework’s incremental contributions. Each row reports the standalone classification performance of a single validation index, demonstrating that no individual component alone approaches the accuracy of the full fusion: the strongest individual contributor (Morphology Flag, 90.4%, 95% CI [89.37, 91.43]) falls 7.7 percentage points below the global framework result (98.13%, 95% CI [97.40, 98.86]), confirming that the performance gain is attributable to the complementary fusion of hardware and software indices rather than any single dominant criterion. For each validation metric, 95% confidence intervals (**CI**) were computed across the 20 participant datasets using the **t-distribution** with 19 degrees of freedom (t = 2.093), following the standard formula:

**CI = mean ± t × (SD/√n)**

The motion flag achieved 84.32% ± 2.71% accuracy (range: 80.00–89.78%), while the impedance flag demonstrated 77.82% ± 5.38% accuracy (range: 68.55–85.97%). These hardware-level indicators enable early rejection of severely corrupted beats, reducing computational burden for downstream processing.

The RR interval plausibility flag achieved 74.52% ± 3.70% with the lowest inter-dataset variability. The DTW morphology flag demonstrated the highest individual accuracy of 90.24% ± 2.06% (Figure 4 and Figure 5 show representative DTW alignments). The SNR flag achieved 82.14% ± 3.25% accuracy, and the baseline wander flag demonstrated 85.83% ± 1.63% accuracy.

**Figure 8:**
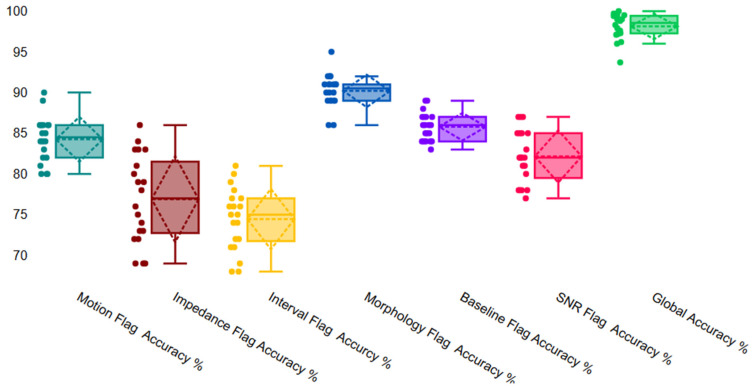
Boxplot of the accuracy of each validation step and the final global accuracy.

Combining all six indices significantly improved performance compared to individual metrics. The global framework achieved 98.13% ± 1.57% mean accuracy (range: 93.68–100%), representing a 7.89 percentage point improvement over the best individual metric (DTW morphology). Five of the twenty datasets (25%) achieved perfect 100% accuracy. Figure 8 summarizes the comprehensive performance metrics and provides detailed visualizations of the accuracy distributions.

**Figure 9:**
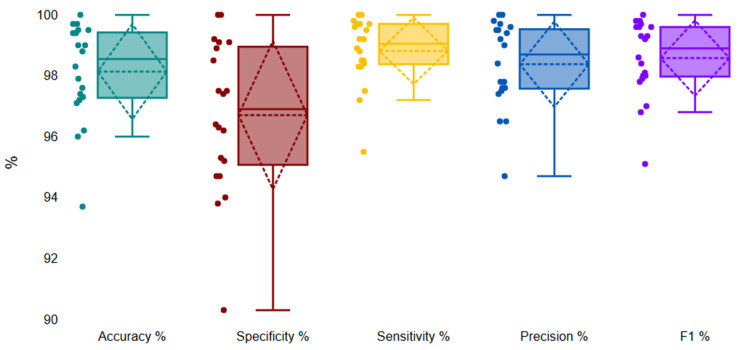
Boxplot of the performance of the final global accuracy.

**Figure 10:**
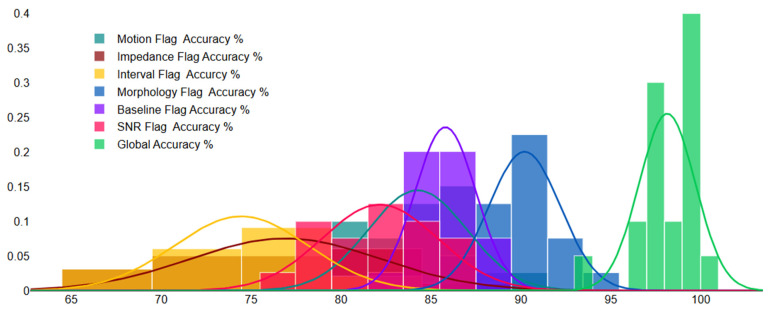
Histogram of the performance matrix of the final global accuracy.

The validation framework was evaluated using standard classification performance metrics including accuracy, sensitivity (recall), specificity, precision (positive predictive value), and F1-score. For each dataset, beats were classified as true positives (Tp: correctly identified acceptable beats), true negatives (Tn: correctly identified unacceptable beats), false positives (Fp: unacceptable beats incorrectly classified as acceptable), and false negatives (Fn: acceptable beats incorrectly classified as unacceptable). Performance metrics were computed individually for the six quality indices to evaluate the global validation outcome after sequential application of both validation stages. The global validation outcome is visualized in the boxplot (Figure 9) and the performance histogram (Figure 10).

The framework demonstrated high sensitivity (98.81%), ensuring minimal rejection of valid beats, and strong specificity (96.70%), confirming robust identification of corrupted beats. The narrow standard deviation indicates consistent performance across diverse participants and recording conditions.

**Figure 11:**
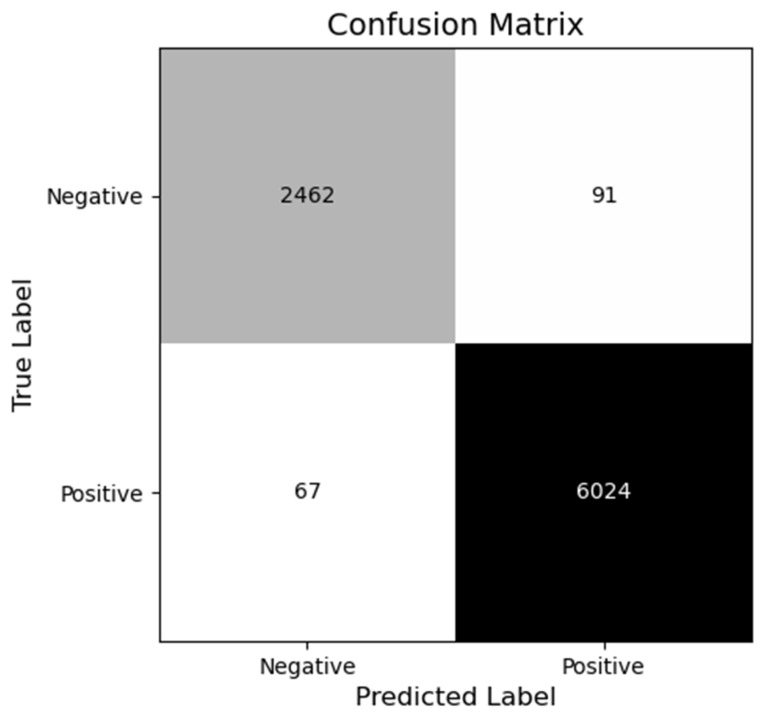
Global Confusion matrix of the final global accuracy.

Figure 11 presents the aggregated confusion matrix. Across all 8644 beats, the framework achieved 6024 true positives, 2462 true negatives, 91 false positives, and 67 false negatives.

These results validate the two-stage hybrid architecture’s effectiveness. The synergistic integration of hardware and software validation indices achieved high performance (98.13% accuracy), substantially outperforming individual metrics while maintaining low false positive rates essential for high-quality AI training dataset curation.

## 6. Performance Comparison with State-of-the-Art Methods

Table 4 presents a contextual performance comparison of the proposed framework against six published ECG signal quality assessment methods, including simple heuristic rules [11,22], machine learning approaches [23,24], and deep learning approaches (CNN, LSTM) [25,26]. The performance values for the benchmark methods (Orphanidou [11], Behar [23], Liu [24], Zhou [25], and Fu [26]) were adopted from the cross-method evaluation reported by Fotsing Kuetche et al. [22], who reimplemented each method and evaluated all of them on three independent datasets under standardized conditions. The values in Table 4 represent the averages across these three datasets. Because the evaluation was conducted on datasets different from those used in the respective original publications, the reported values may differ from the originally published figures. For instance, Orphanidou et al. [11] reported approximately 94% sensitivity and 97% specificity on their own dataset, whereas the reimplementation by Fotsing Kuetche et al. yielded 99.70% sensitivity but only 57.94% specificity when averaged across their three evaluation datasets, reflecting sensitivity to dataset characteristics and class distributions. The proposed framework was evaluated exclusively on the authors’ own dataset and was not included in the Fotsing Kuetche et al. evaluation. Consequently, the comparison serves to contextualize the proposed framework’s performance within the broader ECG quality assessment literature rather than to constitute a controlled benchmark under identical conditions.

**Figure 12:**
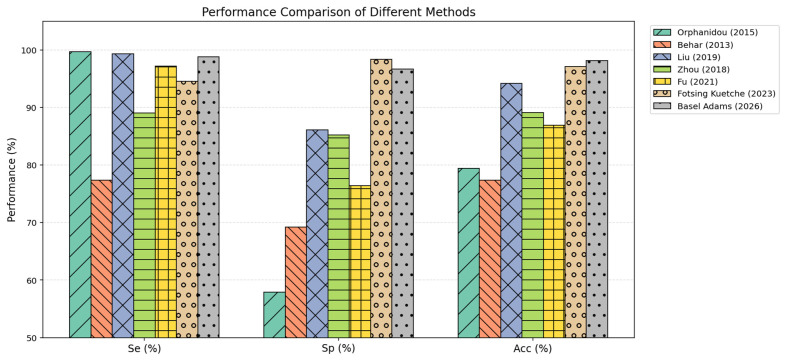
Performance comparison with state-of-the-art methods.

The proposed framework achieved the highest overall accuracy among all evaluated methods (98.13%) as presented in Figure 12. Compared to the Fotsing Kuetche et al. baseline, the framework demonstrated +4.22 percentage points improvement in sensitivity (98.81% vs. 94.59%) while maintaining competitive specificity (96.70% vs. 98.38%). The framework achieved optimal sensitivity-specificity balance, avoiding the tradeoff observed in existing methods where high sensitivity (Orphanidou: 99.70%, Liu: 99.36%) corresponds to substantially lower specificity (57.94% and 86.10%, respectively).

The framework outperformed machine learning methods (Behar: 77.37%, Liu: 94.17%) and deep learning approaches (Zhou: 89.12%, Fu: 86.90%) by 3.96–20.76 percentage points in overall accuracy.

Quantitative validation in this study is restricted to ECG data. Preliminary testing on limited PPG, ICG, and EEG recordings confirmed that the framework’s modular architecture supports modality-specific parameter adaptation; however, these observations are based on small sample sizes without statistical relevance and do not constitute validated applicability. Systematic cross-modality quantitative validation is precluded by the intensive manual annotation effort required to label thousands of beats per biosignal type and remains a priority for future work. Full empirical validation across PPG, ICG, PCG, EMG, and EEG remains a priority for future work as scalable annotation methodologies mature. Additionally, the validation dataset consisted exclusively of healthy participants without known cardiac pathology.

## 7. Discussion

The experimental validation demonstrates that the proposed two-stage hybrid framework achieved strong classification performance on the study dataset (98.13% accuracy, 98.81% sensitivity, 96.70% specificity), comparing favourably with published ECG quality assessment methods under the caveat that no unified benchmarking protocol was applied.

A critical implementation requirement is the synchronized acquisition of IMU and electrode impedance data alongside the biosignal. The IMU sensor must be positioned near the electrodes to accurately detect motion artifacts, and impedance measurement capability is necessary to monitor electrode-skin contact integrity. This hardware prerequisite may not be satisfied by legacy ECG recording systems or minimalist wearable devices lacking auxiliary sensors. For such systems, the framework can operate in software-only mode by bypassing Stage I validation, though with reduced performance. The modular architecture enables graceful degradation when hardware signals are unavailable.

In real-time filtering or validation deployments, it is strongly recommended to forward only beats that successfully pass Stage I hardware gating to Stage II software-based analysis. This strategy provides hard rejection of severely corrupted segments (motion artifacts, electrode detachment) before computationally intensive software validation, substantially reducing processing burden. For resource-constrained wearable platforms, this two-stage filtering approach enables practical real-time implementation by avoiding unnecessary DTW alignment and frequency domain analysis on fundamentally unusable beats.

The computational complexity of frequency-domain SNR analysis and DTW-based morphology assessment may challenge real-time implementation on resource-constrained embedded processors. While Stage I hardware gating substantially reduces the number of beats requiring exhaustive Stage II analysis, optimization through efficient FFT implementations, approximate DTW algorithms, or dedicated hardware acceleration may be necessary for deployment on ultra-low-power wearable platforms. The modular architecture enables selective disabling computationally expensive indices for latency-critical applications where reduced accuracy is acceptable in exchange for lower processing requirements.

Pathological cardiac rhythms, including atrial fibrillation, premature ventricular contractions (PVCs), ventricular tachycardia, and other conduction abnormalities, inherently exhibit irregular R–R intervals. Applying strict interval-based plausibility thresholds in such contexts would risk rejecting clinically meaningful arrhythmic events and biasing subsequent diagnostic analysis. Therefore, in arrhythmia-oriented applications, R–R interval–based rejection criteria are disabled within the signal quality framework. Instead, physiological plausibility validation is restricted to the evaluation of R-peak amplitude stability, which reflects signal integrity without penalizing pathological rhythm dynamics. Similarly, RR interval and amplitudes analysis is not applicable to non-cardiac biosignals such as EMG or EEG, where beat-to-beat interval concepts are undefined.

DTW-based morphological validation is primarily suited for cardiac pulse-like biosignals (ECG, PPG, ICG), where individual beats form meaningful repeatable units. For non-beat-centric biosignals such as EMG or EEG, waveform morphology is not strictly repetitive on a beat-by-beat basis and may exhibit complex nonstationary dynamics. In such cases, template-based DTW validation should be skipped or replaced with modality-specific artifact detection techniques (e.g., spectral quality metrics, independent component analysis, or event-based segmentation). Furthermore, when the validated data will be used for rhythm detection or classification, it is essential to include templates for all possible cardiac rhythms in the multi-dimensional DTW analysis to avoid false rejection of valid pathological waveforms as corrupted beats.

While this population exhibits diverse physiological variability and artifact conditions representative of free-living monitoring, prospective validation on patient populations with structural heart disease, conduction abnormalities, or implanted cardiac devices would strengthen generalizability claims. The narrow inter-dataset performance variability (SD = 1.57%) suggests robust generalization, but clinical validation remains necessary before deployment in diagnostic applications. Although the framework is rule-based rather than learned, the empirical calibration of threshold parameters and fusion weights introduces potential sensitivity to the calibration dataset. While calibration and evaluation were strictly separated at the participant level (Section 3.5), both datasets were drawn from healthy participants recorded under identical hardware and protocol conditions. The calibration subset comprised only three participants (900 beats), which may not fully capture the inter-subject variability present in broader populations. Future work should include recalibration on larger and more diverse cohorts to reduce potential residual variability in the selected parameter values. Independent recalibration and validation on external datasets with different hardware platforms, electrode configurations, and patient populations remain necessary to confirm the generalizability of the selected parameter values.

A further limitation is that the framework was validated exclusively on an internally collected dataset. No external or publicly available dataset was used for independent validation, which limits the generalizability of the reported performance to the specific hardware platform, electrode configuration, and participant demographics employed in this study. This constraint arises primarily from the framework’s requirement for synchronized biosignal, IMU, and electrode impedance recordings, a combination not currently available in public repositories such as PhysioNet. Future work will prioritize evaluation on independent cohorts and, where feasible, publicly available ECG quality benchmark datasets operating in software-only fallback mode (Stage II only) when auxiliary sensor channels are unavailable.

Despite these limitations, the proposed framework successfully addresses fundamental challenges in biosignal quality assessment by achieving optimal sensitivity-specificity balance. The modular two-stage architecture enables application-specific customization while maintaining interpretable validation logic suitable for both real-time monitoring and offline dataset curation.

## 8. Conclusions

This paper presents a two-stage hybrid signal quality validation framework that integrates hardware-based sensor integrity gating with multi-dimensional software quality assessment, quantitatively validated only on ECG and architecturally extensible to additional biosignal modalities pending future statistically powered validation. Unlike prior software-only or modality-specific approaches, the proposed architecture employs synchronized IMU and electrode impedance measurements as prerequisite gating mechanisms before applying computationally intensive software validation, enabling early rejection of irrecoverably corrupted beats while maintaining process efficiency.

Methodological validation on 8644 expert-annotated cardiac beats from 20 participants demonstrated state-of-the-artsignal quality discrimination performance within a healthy cohort: 98.13% accuracy, 98.81% sensitivity, and 96.70% specificity. Prospective clinical validation on patient populations with documented cardiovascular pathology is identified as a required next step before deployment in diagnostic applications. The framework achieved favorable sensitivity–specificity balance relative to six published benchmark methods, though this comparison is contextual rather than controlled as each method was evaluated under different experimental conditions. The narrow inter-dataset variability (SD = 1.57%) and low false positive rate (1.05%) support its suitability for high-purity dataset curation for AI model training. The synergistic integration of complementary validation stages produced 7.89 percentage point improvement over the best individual metric, confirming that hardware and software quality indicators capture orthogonal aspects of signal corruption.

Future work will focus on three directions: first, extension to additional biosignal modalities (EMG, EEG, PCG, respiration) to validate the framework’s modularity across non-cardiac periodic signals; second, machine learning-based optimization of weight parameters and quality thresholds using supervised learning methods (SVM, Random Forest) and neural networks (CNN, LSTM, GRU) trained on expert-annotated datasets, potentially improving discrimination while maintaining interpretability; and third, prospective clinical validation on patient populations with documented cardiac pathology to establish generalization across the full spectrum of cardiovascular disease. In addition, future work will extend the proposed assessment framework by incorporating additional statistical signal quality measures, including kurtosis, skewness, and entropy-based indices, to further enhance robustness against diverse artifact conditions.

The proposed framework provides a robust, interpretable, and computationally efficient solution for both real-time wearable monitoring and offline dataset curation, with direct implications for improving diagnostic accuracy, reducing false alarms in continuous cardiovascular monitoring, and providing a mechanism to improve dataset quality for downstream AI models.

## Figures and Tables

**Table 1:** Fusion weights applied in this study.

Index	Weight	Rationale
**w1: Motion**	1.00	Primary corruption source in wearable ECG
**w2: Impedance**	0.75	Essential for contact integrity; lower individual accuracy
**w3: Interval**	0.50	Lowest individual accuracy; disabled in arrhythmia mode
**w4: Morphology**	1.25	Strongest individual discriminator
**w5: SNR**	0.75	Captures broadband noise contamination
**w6: Baseline**	0.75	Captures low-frequency drift
**Sum**	5.00	

**Table 2:** Framework threshold parameters, values, and selection basis.

Parameter	Value Used	Selection Basis
**τ_motion (IMU variance threshold)**	0.05 m^2^/s^4^ or (0.0005 g^2^)	Empirically calibrated on held-out subset; consistent with motion artifact thresholds reported in [2]
**Z_min (electrode impedance upper bound)**	10 kΩ	AFE hardware specification and standard electrode contact guidelines per Webster [3]
**RR_min/RR_max (physiological HR bounds)**	240 ms/1200 ms (50–250 bpm)	Established physiological heart rate range for wearable ECG monitoring per Clifford et al. [5]
**RR deviation tolerance**	0.5 × **RR_median**	Standard relative tolerance used in ambulatory ECG quality frameworks; consistent with [5,11]
**k (DTW sigma multiplier)**	2.0	Empirically calibrated on held-out subset; k = 2 corresponds to the 95th percentile of the DTW distance distribution under clean signal conditions
**θ_DTW**	μ + 2σ of clean-beat DTW distribution	Derived per-participant from the held-out calibration subset using the clean-beat DTW distance distribution
**θ_SNR**	10 dB	Lower bound of the acceptable monitoring quality range consistent with ambulatory ECG SNR literature [10,11]
**θ_baseline**	0.75	Empirically calibrated on held-out subset; corresponds to a maximum tolerated baseline power contribution of 25% of total signal power

**Table 3:** Performance statistics for all stages.

Accuracy Metric	Mean (%)	±SD (%)	Min (%)	Max (%)	95% CI
**Motion Flag**	84.4	2.7	80	90	[83.14, 85.66]
**Impedance Flag**	77.7	5.3	69	86	[75.22, 80.18]
**Interval Flag**	74.8	3.8	68	81	[73.02, 76.58]
**Morphology Flag**	90.4	2.2	86	95	[89.37, 91.43]
**SNR Flag**	82.2	3.2	77	87	[80.70, 83.70]
**Baseline Flag**	85.9	1.9	83	89	[85.01, 86.79]
**Global Validation**	98.1	1.5	93.6	100	[97.40, 98.86]

**Table 4:** Performance comparison.

Method	Evaluation	Sensitivity (%)	Specificity (%)	Accuracy (%)
Orphanidou [11]	Set of Rules +average template matching	99.70	57.94	79.41
Fotsing Kuetche [22]	Set of Rules + beats correlation and clustering	94.59	98.38	97.10
Behar [23]	SVM	77.34	69.20	77.37
Liu [24]	SVM	99.36	86.10	94.17
Zhou [25]	CNN	89.02	85.23	89.12
Fu [26]	LSTM	97.19	76.42	86.90
Basel Adams	Sensor Fusion and Software Indices	98.81	96.70	98.13

## Data Availability

The original contributions presented in this study are included in the article. Further inquiries can be directed to the corresponding author. The data presented in this study are not publicly available due to restrictions imposed by the ethics approval under which the study was conducted. The storage and sharing of participant data is limited by the terms of the ethical approval, and therefore the data cannot be made publicly accessible.
